# Reconstructing dynamics of foodborne disease outbreaks in the US cattle market from monitoring data

**DOI:** 10.1371/journal.pone.0245867

**Published:** 2021-01-27

**Authors:** Ray Huffaker, Monika Hartmann

**Affiliations:** 1 Department of Agricultural and Biological Engineering, University of Florida, Gainesville, Florida, United States of America; 2 Institute for Food and Resource Economics, University of Bonn, Bonn, Germany; US Department of Agriculture, UNITED STATES

## Abstract

Conventional empirical studies of foodborne-disease outbreaks (FDOs) in agricultural markets are linear-stochastic formulations hardwiring a world in which markets self-correct in response to external random shocks including FDOs. These formulations were unequipped to establish whether FDOs cause market reaction, or whether markets endogenously propagate outbreaks. We applied nonlinear time series analysis (NLTS) to reconstruct annual dynamics of FDOs in US cattle markets from CDC outbreak data, live cattle futures market prices, and USDA cattle inventories from 1967–2018, and used reconstructed dynamics to detect causality. Reconstructed deterministic nonlinear market dynamics are endogenously unstable—not self-correcting, and cattle inventories drive futures prices and FDOs attributed to beef in temporal patterns linked to a multi-decadal cattle cycle undetected in daily/weekly price movements investigated previously. Benchmarking real-world dynamics with NLTS offers more informative and credible empirical modeling at the convergence of natural and economic sciences.

## Introduction

Foodborne diseases impose substantial economic burden worldwide. In the US, there are approximately 48 million foodborne illnesses each year costing over $15 billion annually [1]. Twenty percent of these illnesses (9.4 million) is attributed to known bacterial, parasitic, and viral pathogens [1]. Depending on the pathogen, symptoms of foodborne illness to consumers may be severe—including bloody diarrhea, fever, severe stomach pain, vomiting, and kidney failure—and result in hospitalization and even death.

Past empirical economic models have investigated the reaction of agricultural markets to foodborne disease outbreaks (FDOs) with a variety of linear-stochastic specifications [2–7] used in “most empirical investigations of agricultural markets”([8], p. 114). In conformity to dominant theory, linear modeling hardwires stable agricultural markets that equilibrate as rational agents process all available information to re-adjust supply and demand in response to exogenous random FDO shocks. Short-term net price impacts are expected to be negative as negative price impacts of downward shifts in demand for contaminated food dominate positive price impacts of inward shifts in supply (e.g., from government recalls) [2]. Eventually, agricultural markets are expected to stabilize (‘self-correct’), so past work searched for downward price spikes from when an FDO shock occurred to when the market returned to ‘normal’ levels [5].

Lusk and Schroeder (2002) attributed the inability of “our models [to] detect the market reaction” (p. 58) to inadequate representation of market dynamics; in particular, the “[possibility] that the impacts of recalls on meat demand are more cumulative in nature and gradually reduce market demand over time instead of causing notable short-run declines” (p. 57). They conjectured that “if there is any systematic change in cattle and hog demand due to recalls, it likely occurs over an extended period of time” (p. 47). They further conjectured that “futures markets may not react to meat recalls because…the market has already incorporated this information” (p. 57). Futures prices factor in expected movements in spot prices (payments for immediate delivery of a commodity). Downward price spikes would not be detected if traders had already factored in expected negative market consequences of FDOs and were forecasting economic recoveries.

In this paper, we empirically map out causal interactions between agricultural market variables and FDOs applying recently-developed data-driven methods in the science and mathematical statistics literature. We extend past work that relied on regression techniques to measure marginal relationships among variables. Regression coefficients do not imply causality, but rather *cum hoc non propter hoc* (`with this, not because of this’). Selecting an appropriate causal detection method depends critically on the nature of underlying system dynamics [9]. The popular Granger causality-detection method [10] is designed for use with linear-stochastic systems like those conventionally used to model agricultural markets. Linear-stochastic systems impose linear separability among variables, which implies that “information about a causative factor is independently unique to that variable (e.g., information about predator effects is not contained in time series for the prey), and can be removed by eliminating that variable from the model” [9]. Consequently, variable *X* Granger-causes *Y* if the predictability of *Y* decreases when *X* is removed from the set of possible causal factors.

However, Sugihara et al. (2012) demonstrated that Granger causality gives unreliable results when system dynamics are not linearly separable. In *weakly-coupled* systems, deterministic nonlinear interactions encode information about *X* into *Y*, and this information does not disappear from *Y* when *X* is removed from the system. As noted by the famous naturalist John Muir (1911), “When we try to pick something up by itself, we find it hitched to everything else in the universe” [11]. Sugihara et al. (2012) developed the *Convergent Cross Mapping* (CCM) method to detect causal networks in real-world systems diagnosed with deterministic nonlinear dynamics.

Deterministic nonlinear dynamics offer a possible alternative approach for capturing the cumulative systematic interactions between agricultural market variables and FDOs proposed by Lusk and Schroeder (2002). Chavas and Holt (1993) questioned reflex reliance on self-correcting linear agricultural market models in light of emerging results demonstrating that instability can emerge endogenously from nonlinear dynamic systems [12, 13]. Agricultural markets need not self-correct to ‘normal’ levels due to destabilizing endogenous factors including highly inelastic demands [8, 14]; nonlinear cobweb price expectations [15]; and financial, institutional, and biophysical constraints frustrating supply from matching demand [16]. *The Economist* recently recommended that “like physicists, [economists] should study instability instead of assuming that economies naturally self-correct” [17].

Consequently, our first task is to select the appropriate empirical causal-detection method by diagnosing whether observational data on agricultural marketing variables and FDOs are most likely generated by linear-stochastic or deterministic-nonlinear system dynamics. We make this diagnosis with *Nonlinear Time Series Analysis* (NLTS)—an inductive science approach in which scientists “are presented with observations and asked… to go backward to solve for [the system] that made them”([18], p. 110). NLTS is designed to empirically reconstruct real-world *phase-space* dynamics from the observational data they generate without prior knowledge of system equations [19–21].

In a nutshell, phase space is the graphical portrayal of system dynamics. Phase space coordinates are provided by system variables. Each *n*-dimensional point in phase space records the levels (states) of *n* system variables at a point in time. Phase space trajectories connecting these points depict the co-evolution of system variables from given initial states. If system dynamics are *dissipative*, trajectories converge toward an attractor–a geometric object bounded within an *m*-dimensional subset of *n*-dimensional phase space (*m*<<*n*). Once a trajectory evolves along an attractor, it never escapes [22]. Consequently, dissipative dynamics may be dimension-reducing since long-term behavior occurs wholly within a reduced *m*-dimensional subspace. The problem of modeling system dynamics shrinks by the *n*-*m* inactive dimensions [23].

To apply NLTS, we proceed as if system variables are weakly coupled, and then statistically test the veracity of this assumption at a later stage. This assumption allows us to reconstruct phase-space dynamics from even a single time series variable with *delay-coordinate embedding*, in which time-delayed copies of an observed variable provide surrogates for omitted but weakly-coupled covariates [24]. An attractor reconstructed in delay-coordinate phase space is called a *shadow* attractor. *Takens* theorem states conditions guaranteeing that delay-coordinate embedding is a one-to-one mapping of points from the shadow to the original attractor, and thus preserves essential mathematical properties [24]. We use *surrogate data* [25] to statistically test whether apparent geometric regularity in a reconstructed shadow attractor is most likely systematically generated by deterministic-nonlinear dynamics (indicating CCM causality detection), or fortuitously generated by linear-stochastic dynamics (indicating Granger causality detection).

Following past studies [2–4], we focus on live cattle futures prices traded on the Chicago Mercantile Exchange as a market performance variable embedding national expectations of how supply and demand adjust to FDOs attributed to beef. Live cattle are ‘finished’ products that have reached the necessary weight for processing [26]. Supply and demand factors for beef typically play the biggest role in determining live cattle prices. We include the annual inventory of US cattle on feed touted by commodities brokerage firms to be a key indicator of future live cattle supply [26].

Past work represents FDOs attributed to beef indirectly with indices weighing the length and severity of government recalls of contaminated meat. Alternatively, we represent FDOs attributed to beef with actual reported outbreaks collected on the Foodborne Disease Outbreak Surveillance System (FDOSS) by the Centers for Disease Control and Prevention (CDC). We discuss these time series in detail below.

As reported below, our NLTS results show that observed data on the above-mentioned variables are most likely generated by deterministic-nonlinear food system dynamics. Consequently, we applied CCM to test for the following causal interactions: Do FDOs attributed to beef cause a market response in live cattle futures prices? Do beef market variables drive FDOs attributed to beef? In other words, do beef markets propagate their own outbreaks? We might well expect this given that foodborne infections are transmitted systematically from ‘farm to fork’ along food supply chains [27].

## Data

The overlapping period of record for FDOs attributed to beef (B-FDO), the cattle inventory (CI), and live cattle futures prices (LCFprices) is 1967–2018 (52 observations).

### FDOs attributed to beef

The Centers for Disease Control and Prevention (CDC) collect national time series records on FDOs reported by state public health agencies with the Foodborne Disease Outbreak Surveillance System (FDOSS). FDOSS defines FDOs as “the occurrence of two or more cases of a similar illness resulting from consuming a common food.” In annual reports available from 1967–2016, ‘total’ outbreaks (‘confirmed’ and ‘unconfirmed’) are differentiated among food groups and summed over etiologies (i.e., bacterial, parasitic, viral, and unknown pathogens). We downloaded these from the National Outbreak Reporting System (NORS) Dashboard (https://www.cdc.gov/fdoss/annual-reports/index.html). NORS provides a web-based platform (launched in 2009) that local, state, and territorial health departments in the U.S. use to report FDOs and other types of infectious disease outbreaks (https://www.cdc.gov/nor/). Annual CDC reports are not available for the years 2003–2005 and 2009–2010. To fill in missing years, and extend the time-series record to 2018, we used annual counts of national FDOs maintained in parallel on the NORS Dashboard starting in 1998 (https://wwwn.cdc.gov/norsdashboard/). In results reported below, we found that this combination of sources did not create a nonstationary record that would have signaled abrupt shifts in dynamic behavior.

### Live cattle futures prices

Analysts conventionally convert a stream of heterogeneous futures contracts into a *continuous futures contract history* for purposes of time series analysis and back-testing of investment strategies. The computation is based on popular algorithmic components including *front-month contracts*, *first-of-month roll dates*, and *calendar-weighted adjusted prices*. Front-month contracts have expiration dates closest to the current calendar date. First-of-month roll dates allow traders to ‘roll over’ front-month contracts to contracts with later expiration dates. Calendar-weighted adjustment takes a weighted average of contract prices over a window around the roll date [28]. We use publicly-available live cattle futures (average annual settle prices) for *CME live cattle futures #1 contracts* computed by the MacroTrends group available at https://www.macrotrends.net/futures/cattle. We followed convention [29] in correcting these prices for inflation with the US CPI available at https://fred.stlouisfed.org.

### Cattle inventory

The United States Department of Agriculture (USDA) provides the annual cattle inventory (i.e., cattle and calves on feed January 1 for slaughter market in US from feedlots with capacity of 1000 or more head) in reports available at https://usda.library.cornell.edu/concern/publications/m326m174z?locale=en&page=59#release-items.

### NLTS methods

We outline a five-stage procedure for reconstructing B-FDO dynamics in the US cattle market from monitoring data with NLTS methods, and provide more detailed discussion of each method in an appendix. NLTS methods are covered in depth in original papers cited in the text, application papers [30–35], and introductory books [13, 19, 20].

In Stage 1, we apply *Singular Spectrum Analysis* (SSA) [36] to decompose each standardized time-series record into structured variation (signal)—composed of low-frequency trend and nonlinear trend cycles, and higher-frequency oscillatory components—and unstructured variation (noise) [36, 37]. SSA measures signal strength as the portion of total variance in a record explained by the signal, and thus gives an initial indication of whether there is adequate structure to merit further search for deterministic-nonlinear structure with NLTS. In Stage 2, we apply *Singular Spectrum Transformation* (SST) [38] to screen strong signals for *change-points* indicating abrupt shifts in dynamic structure during the period-of-record that violate nonlinear stationarity required by NLTS. In Stage 3, we test strong stationary signals for deterministic-nonlinear dynamic structure. We reconstruct shadow phase space from each signal with time- delay embedding, which uses delayed copies of a signal as surrogates for other variables. We use surrogate data to test whether apparent geometric regularity in a shadow attractor is generated fortuitously by linear-stochastic dynamics as opposed to deterministic nonlinear dynamics [25, 39]. In Stage 4, we apply *Convergent Cross Mapping* [9] (CCM) to detect pairwise causal interactions among signals evincing deterministic nonlinear dynamic structure. The logic behind CCM is that shadow attractors reconstructed from interacting variables map one-to-one with the same real-world attractor, and thus map one-to-one with each other. We further apply *Extended-CCM* [40] to screen detected interactions for false positives in which synchronized behavior is mistaken for causal interaction. In Stage 5, we apply the *S-Mapping* method [21] to quantify interactions identified with CCM as partial derivatives measuring marginal changes in a response variable to incremental increases in a driving variable.

## Results

### Signal processing

**Fig 1:**
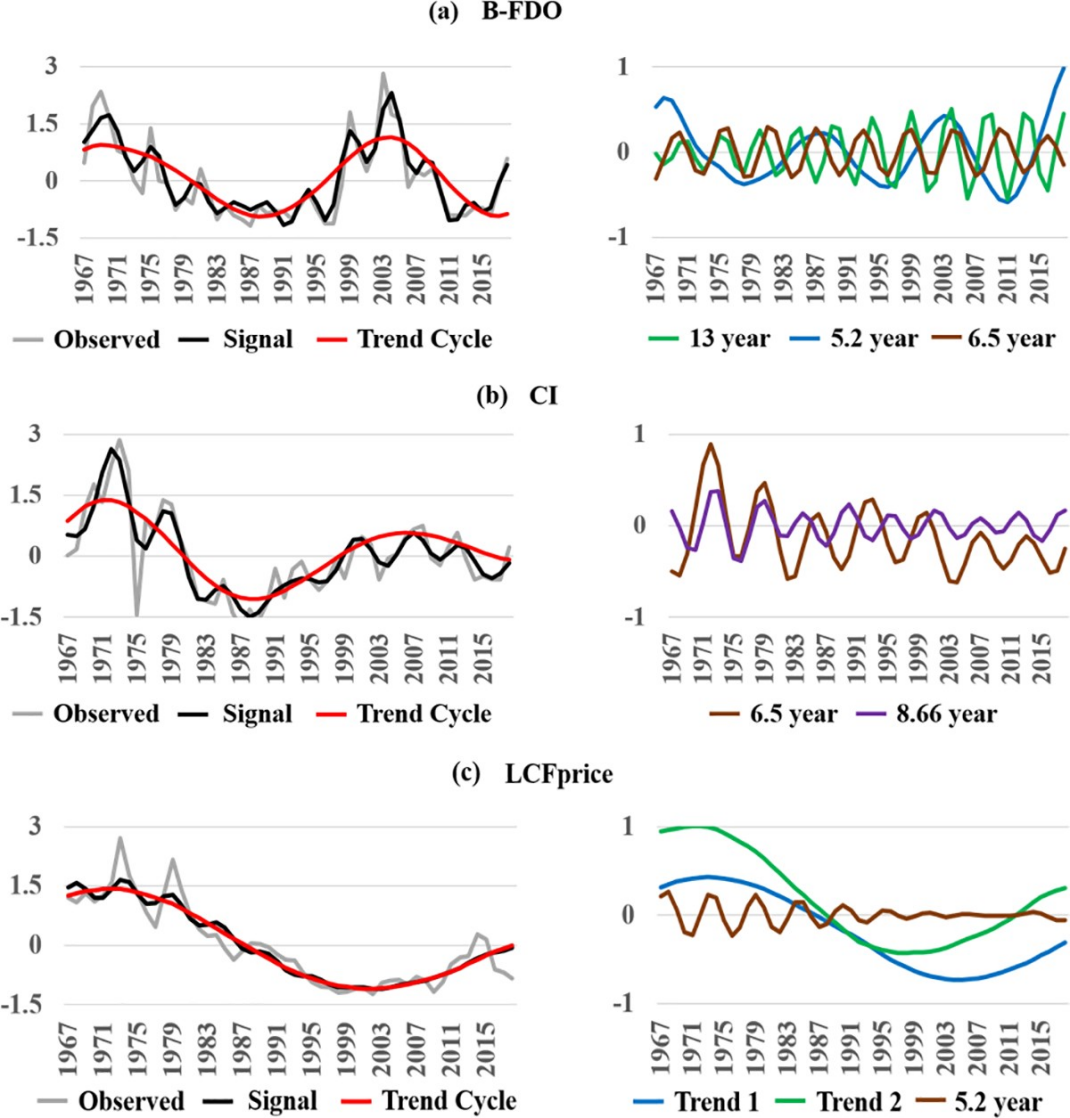
Singular Spectrum Analysis (SSA) of standardized time-series records. Leftward plots show the observed record (grey curve), and the isolated signal (black curve) and low-frequency nonlinear trend components (red curve) for each record. Rightward plots show higher-frequency oscillations. **(a)(b)** The B-FDO signal (foodborne disease outbreaks attributed to beef) and CI signal (cattle inventory) are dominated by strong multi-decadal nonlinear trend cycles accounting for over 50% of total variation, with higher-frequency 5.2- to 13-year cycles also showing appreciable composite signal strength (Table 1). The nonlinear-trend cycles isolated from the B-FDO record and CI record are strikingly similar. **(c)** The LCFprice signal (live cattle futures market prices) is dominated by a multi-decadal nonlinear trend cycle accounting for 86% of total variation with a faint higher-frequency 5.2-year cycle accounting for 4% (Table 1). The multi-decadal nonlinear trend cycle is the sum of two independent trend cycles shown in the rightward panel (blue and green curves).

We standardized each record by subtracting the mean from each observation and dividing by the standard deviation. Standardizing data is conventional practice in time-series analysis to put multiple time series records with different units on a comparable *standard deviation* scale. Fig 1 presents the results of applying SSA in two sets of plots for each record. Leftward plots graph the observed record (grey curve), the signal (black curve) and low-frequency nonlinear trend components (red curve); and rightward plots graph higher-frequency oscillations. In leftward plots, vertical differences between the observed record and the signal measure noise in each year, which may be due to non-systematic measurement error. The records are characterized by strong signals that account for 85%, 81%, and 90% of total variation in the B-FDO, CI, and LCFprice records, respectively (Table 1).

**Table 1:** Signal processing with singular spectrum analysis^a^.

	signal ^c^	nonlinear trend cycle	(4, 6]	(6, 8]	(8, 13]
**B-FDO**	85%	54%	9%	6%	16%
**CI**	81%	58%		18%	5%
**LCFprice** ^**b**^	90%	86%	4%		

^**a**^ B-FDO is annual foodborne disease outbreaks attributed to beef in the US, CI is annual cattle inventory in the US, and LCF is live cattle futures market prices (variables standardized).

^**b**^ LCFprice is deflated by the U.S. CPI.

^**c**^ Signal strength measured as percent of total variation (from the mean) accounted for in the observed record.

Signals isolated from the B-FDO record (Fig 1A) and the CI record (Fig 1B) are dominated by strong multi-decadal nonlinear trend cycles each accounting for at least 54% of total variation, with higher-frequency cycles also showing appreciable composite signal strength (Table 1). The B-FDO multi-decadal trend cycle is strikingly similar to the CI multi-decadal trend cycle. We test below whether this similarity indicates that CI drives B-FDO, B-FDO drives CI, CI and B-FDO are bi-causally interactive, or both are synchronized to an outside force. In Fig 1C (left plot), the signal (black curve) isolated from the discounted LCFprice record (grey curve) is dominated by a multi-decadal nonlinear trend cycle accounting for 86% of total variation with a faint higher-frequency 5.2-year cycle accounting for 4% (Table 1). This multi-decal cycle is the sum of two independent nonlinear trend cycles shown in the rightward panel of Fig 1C (blue and green curves).

In sum, SSA results indicate that each time series has a strong signal comprising substantial structured variation that merits further investigation with NLTS. In particular, B-FDO occurs in regular low- and high-frequency cycles that cast suspicion on their conventional treatment as random shocks to the food system.

### Nonlinear stationarity

Stationarity indicates that the “duration of the measurement is long compared to the time scales of the systems” [38]. Consequently, an important implication of finding the B-FDO, CI, and LCFprice signals to be stationarity is that the period-of-record (1967–2018) is long enough to adequately sample the dominant low-frequency nonlinear trend cycles isolated by SSA.

**Fig 2:**
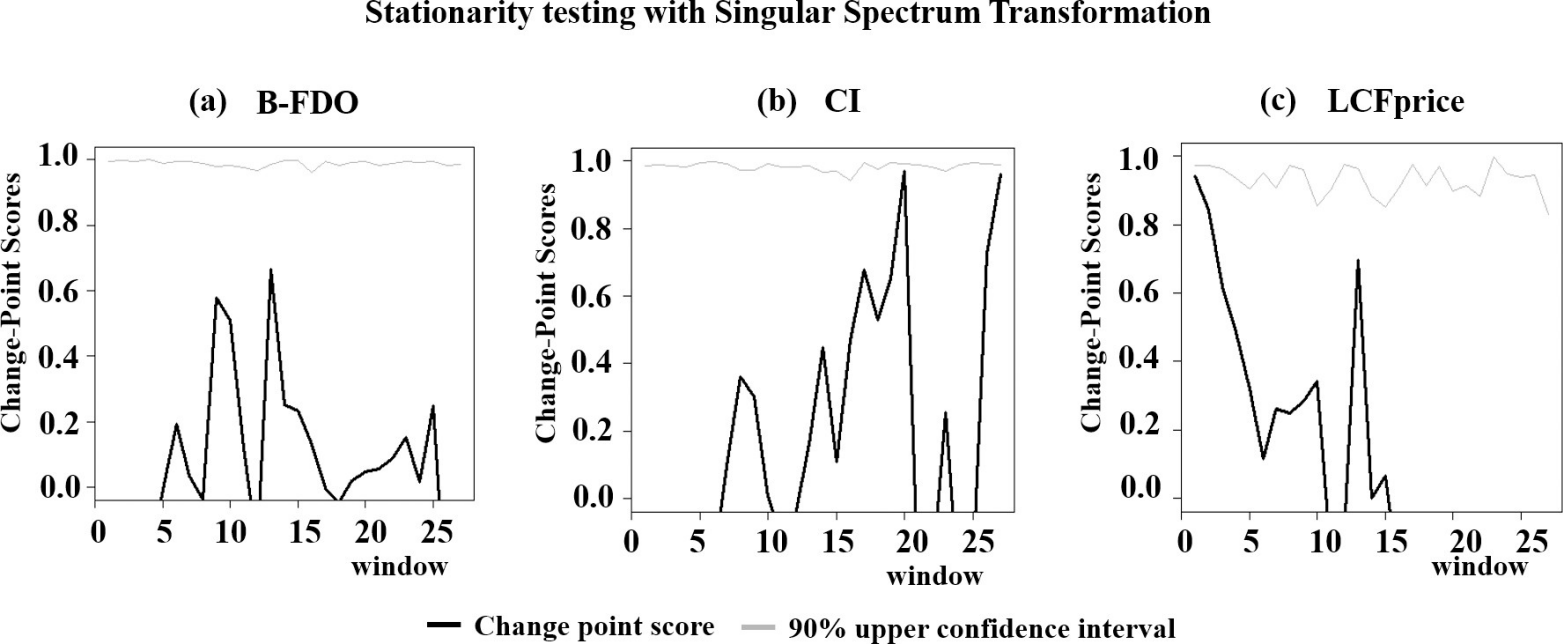
Testing for nonlinear stationarity with Singular Spectrum Transformation (SST). Change-point scores (black curves) resting below upper bootstrapped 90% confidence limits (grey curves) represent statistically insignificant abrupt shifts in dynamic structure violating nonlinear stationarity. SST did not detect significant change points in the B-FDO signal (indicating that the NORS data was compatible with the cycles driving the FDOSS data), the CI signal, or the LCFprice signal with the Trend 2 component removed (Fig 1(C), right panel, green curve).

In Fig 2, we show the results of applying *Singular Spectrum Transformation* (SST) to detect change-point scores representing years in which abrupt structural shifts in dynamic structure occur, and consequent violations of nonlinear stationarity (black curves). Change-point scores are statistically insignificant if they rest below an upper bootstrapped 90% confidence limit (grey curves) generated with randomized surrogate data vectors. SST did not detect statistically significant change points in the B-FDO signal (indicating that the NORS data was compatible with the cycles driving the FDOSS data) or the CI signal. SST detected a change point in the LCFprice signal that disappeared when the weakest nonlinear trend component was removed from the signal, i.e., Trend 2 (Fig 1(C), right panel, green curve). We continued to analyze the stationary partially-detrended LCFprice, which accounts for 55% of total variation in observed LCF prices.

On a final note, the CDC conjectured that the steep upswing in reported total outbreaks in 1998 is a break in the record caused by the adoption of enhanced outbreak surveillance measures (see Annual Report for years 1998–2002). There was also a steep upswing in B-FDO in 1998 (Fig 1A, grey curve). However, as reported above, SST screening did not detect a change point in B-FDO in 1998, indicating that enhanced surveillance by the CDC did not make the B-FDO record nonstationary. A possible contributing endogenous explanation for the upswing in B-FDO in 1998 is that outbreaks were trending upward along with cattle inventories (Fig 1B, red curve).

### Testing for deterministic-nonlinear dynamics

**Fig 3:**
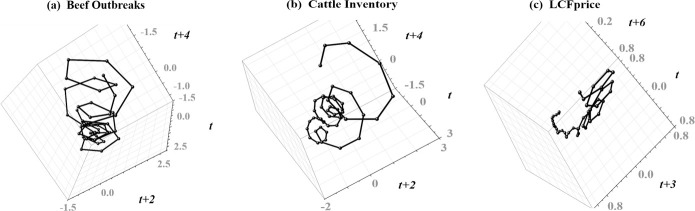
Nonlinear phase space reconstruction with time-delay embedding. Shadow attractors reconstructed from the **(a)** B-FDO signal (annual foodborne disease outbreaks attributed to beef), **(b)** the CI signal (cattle inventory), and **(c)** the LCFprice signal (live cattle futures market prices) with the upward linear trend removed. Each shadow attractor exhibits wide-swinging outer oscillations reflecting multi-decadal nonlinear trend cycles, and tighter interior cycling reflecting higher-frequency oscillations.

We reconstructed shadow attractors from the B-FDO, CI, and partially-detrended LCFprice signals that exhibit substantial geometric regularity reflecting signal cyclical components isolated with SSA (Fig 3A–3C). Each shadow attractor exhibits wide-swinging outer oscillations reflecting multi-decadal nonlinear trend cycles, and tighter interior cycling reflecting higher-frequency oscillations.

We generated *PPS* surrogate data vectors [41] to test the null hypothesis that apparent structure in each shadow attractor is most likely generated by random shifting of a periodic orbit characteristic of noisy linear dynamics [42], and selected nonlinear prediction skill [43] as the discriminating statistic. We ran an upper-tailed test since high nonlinear prediction skill is consistent with deterministic-nonlinear structure, and set a *α* = 95% significance level. Applying nonparametric rank-order statistics [39], we constructed an ensemble of *S* = (*k*/*α*)−1 = 399 surrogate data vectors, and accepted the null hypothesis if the nonlinear predictive skill—measured as Nash-Sutcliffe Model Efficiency (NSE) [44]—taken from a signal attractor did not fall among the top *k* = 20 surrogate values. We rejected the null hypothesis for each signal (Table 2), leaving open the possibility of deterministic-nonlinear dynamics.

**Table 2:** Surrogate data results using rank-order statistics^a^^,^^b^.

B-FDO	Signal ^c^	Surrogate (high) ^d^	H_0_
Prediction Skill	0.83	0.02	Reject
**CI**			
Prediction Skill	0.81	-0.093	Reject
**LCFprice**			
Prediction Skill	0.72	-0.184	Reject

^**a**^ B-FDO is annual foodborne disease outbreaks attributed to beef in the US, CI is annual cattle inventory in the US, and LCF is live cattle futures market prices (variables standardized).

^**b**^ Surrogates are used to test the null hypothesis that aperiodic cycling characterizing the empirically-reconstructed attractors is generated by randomly shifting periodic orbits characteristic of noisy linear dynamics. The significance level is set at *α* = 95% with 399 surrogates generated.

^**c**^ Nonlinear prediction skill measured as Nash-Suttliffe Model Efficiency (NSE).

^**d**^ An upper-tailed test rejects the null hypothesis if the NSE computed using the shadow attractor reconstructed from the signal rests above the floor of the upper extreme values computed from surrogate attractors.

### Causality detection

**Fig 4:**
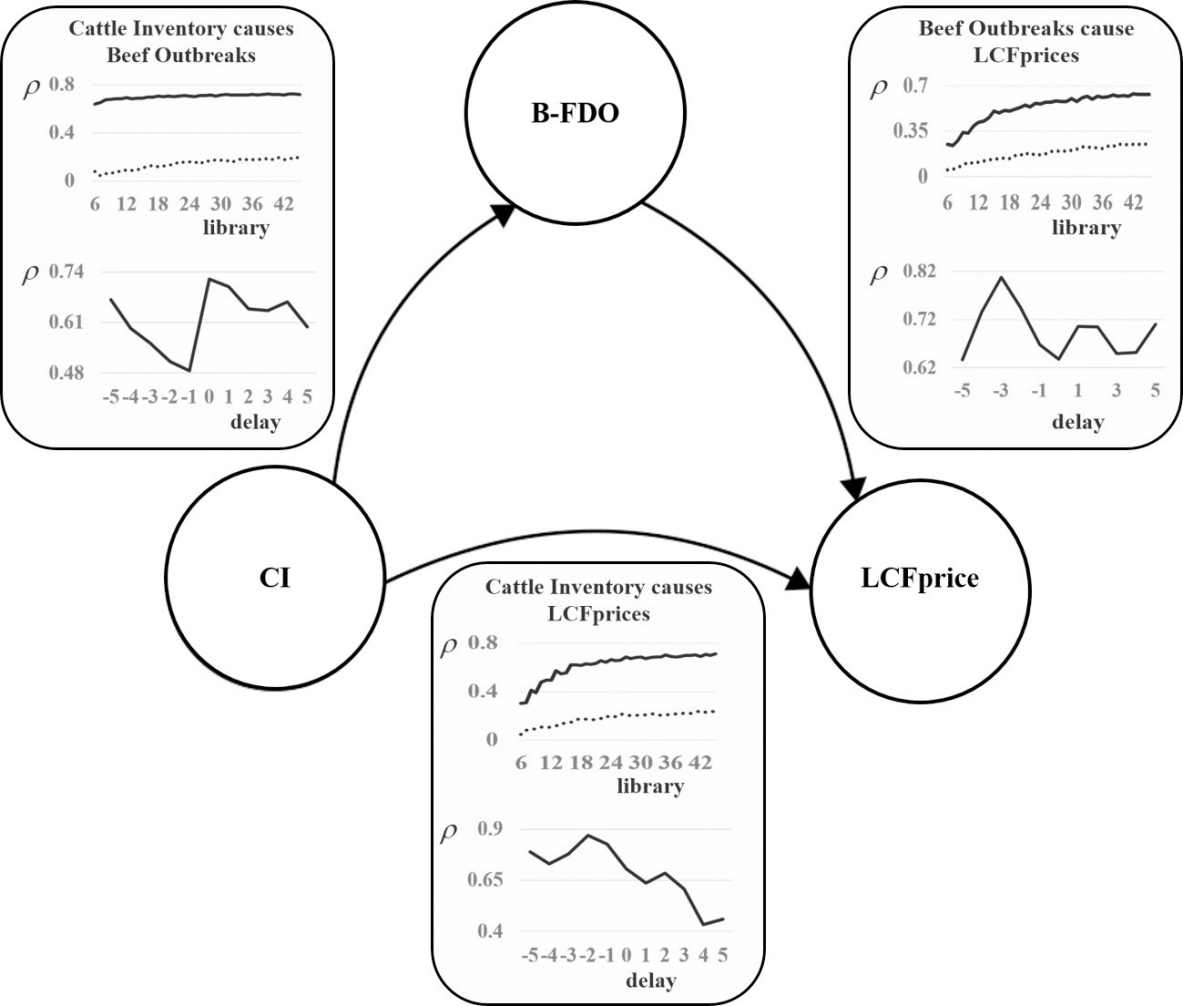
Causality detection. Pairwise causal interactions passing both CCM and Extended-CCM detection are summarized in a causal network diagram in which circular hubs depict interactive signals and incoming arrows denote driving interactions. Evincing market response, LCFprice is driven by both B-FDO and CI. Evincing transmission of FDOs attributed to beef from ‘farm to fork’, CI drives B-FDO. The plots adjacent to each arrow display results of corresponding CCM (top) and Extended-CCM (bottom) tests. Each detected interaction is moderately strong since CCM curves (black curve) converge to a correlation coefficient *ρ* of at least 65%, and statistically significant since it rests above a bootstrapped 95% lower confidence bound (dashed curves). The Extended-CCM curve for each cross mapping peaks at non-positive delays, ruling out false-positive noncausal sychronous interactions.

In Fig 4, we summarize pairwise causal interactions passing both CCM and Extended-CCM detection in a causal network diagram in which circular hubs depict interactive signals and incoming arrows denote driving interactions. In evidence of market response, LCFprice is driven by both B-FDO and CI. In evidence of the transmission of FDOs attributed to beef from ‘farm to fork’, CI drives B-FDO. Adjacent to each arrow is a box with plots showing results of corresponding CCM (top) and Extended-CCM (bottom) tests. Each detected interaction is moderately strong since it is associated with a CCM curve (black curve) that converges to a correlation coefficient *ρ* of at least 65%. Moreover, each CCM curve is statistically significant since it rests above a bootstrapped 95% lower confidence bound (dashed curves) computed with surrogate data as detailed in the appendix. The Extended-CCM curve for each cross mapping peaks at non-positive delays, empirically validating that detected causal events occur before (or contemporaneously with) responses. This rules out false-positive noncausal sychronous interactions.

### Causality quantification

**Fig 5:**
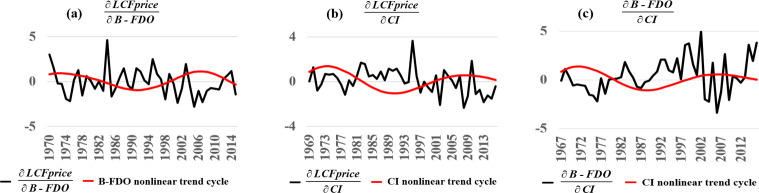
Causality quantification. The S-Mapping method of Deyle et al. (2018) was used to compute partial derivatives measuring the marginal change in a response variable *X* to an incremental change in a driving variable *Y* (∂*X*/∂*Y*) in each year for each interaction in the causal network diagram (Fig 4). Each computed marginal response (black curves) exhibited some regularity depending on the phase of multi-decadal nonlinear trend cycles isolated with SSA (red curves). **(a)** The ∂LCFprice/∂B-FDO response follows a 4-year cycle whose values are mostly positive when standardized B-FDO is below average along the B-FDO multi-decadal cycle (1980–1997), and is erratic when B-FDO is above average along the multi-decadal cycle. **(b)** The ∂LCFprice/∂CI response is predominantly positive (72% of years) when standardized CI is below average along the CI mult-idecadal cycle (1981–1998), and otherwise more erratic. **(c)** The ∂B-FDO/∂CI response is predominantly positive (78% of years) when CI is below average along the multi-decadal CI cycle (1981–1998), and otherwise more erratic.

Fig 5 shows results of using the S-Mapping approach of Deyle et al. (2018) to compute partial derivatives measuring the marginal change in a response variable *X* to an incremental change in a driving variable *Y* (∂*X*/∂*Y*) in each year for each interaction in the causal network diagram (Fig 4). In Fig 5A, the ∂LCFprice/∂B-FDO response (black curve) occurs with a 3-year delay detected by Extended-CCM so that the first response year is 1970. The marginal economic response exhibits some regularity that depends on the phase of the multi-decadal nonlinear trend cycle for B-FDO (red curve). When B-FDO is below average along the cycle (1980–1997), the marginal impact follows a 4-year cycle whose values are positive 59% of the time. Alternatively, when standardized B-FDO is above average along the cycle, the marginal impact of another outbreak on LCFprice is more erratic.

In Fig 5B, the ∂LCFprice/∂CI response (black curve) occurs with a 2-year delay detected by Extended-CCM so that the first response year is 1969. When CI is below average along the nonlinear trend cycle (red curve, 1981–1998), the marginal response of LCFprice to an incremental increase in CI is positive 72% of the time. Alternatively, when standardized CI is above average along the multi-decadal CI nonlinear trend cycle, the marginal impact becomes more erratic.

In Fig 5C, the ∂B-FDO/∂CI response (black curve) has a 0-year delay detected by Extended-CCM so that the initial response year is 1967. When CI is below average along the multidecadal nonlinear trend cycle (red curve, 1981–1998), the marginal impact on B-FDO of an increment in CI is positive 78% of the time. Alternatively, when CI is below average along the cycle, the response is more erratic.

## Discussion

NLTS is an inductive science approach that infers causal structure from observational data, as opposed to a conventional reductionist modeling approach that “assume[s] that we already knew the causal structure in order to make measurements of causal strengths and to conduct counterfactual analysis”([45], p. 37). As such, NLTS provides positive analysis of behavior that ‘actually happened’. Comparing this to what ‘should have happened’ in the judgement of the researcher may well be inaccurate without direct knowledge of the internal business objectives of cattle producers, consumers, and futures traders. Economic agents realizing undesireable outcomes might have avoided the behavior in hindsight. We are limited to speculating a few of many possible explanations for causal behavior uncovered by NLTS diagnostics.

We first diagnosed that observational data on agricultural marketing variables and FDOs are most likely generated by deterministic-nonlinear system dynamics, which directed us to the *Convergent Cross Mapping* (CCM) causal-detection method based on state-space reconstruction techniques. This finding is interesting in and of itself because it offers empirical evidence of the recognized theoretical possibility that real-world agricultural markets can be endogenously unstable and not naturally self-correct to external shocks [8, 33, 46].

We uncovered moderately strong causal interactions among the beef market covariates included in this research:

First, CI drives B-FDO, providing empirical evidence that outbreaks are endogenously generated by the beef market. SSA signal processing of comparable standardized values demonstrated that causality from CI to B-FDO has expressed itself in a multi-decadal trend cycle in CI that is tracked to a surprising degree by a multi-decadal trend cycle in B-FDO (Fig 1A and 1B). Together these results suggest that the multi-decadal CI trend cycle provides a quick and informative predictor of long-term trends in outbreak occurrence based on rigorous empirical causality testing.

That the supply side of the beef market causes its own outbreaks is consistent with documented pathogenic contamination of meat products along slaughter lines, and during processing, storage, and preparation [47]. It is also consistent with economic explanations partially attributing foodborne disease to market failures that weaken the profit incentives of suppliers to invest in greater preventative measures against foodborne pathogens [48, 49]. Similar to ‘lemons’ in used-car markets, consumers cannot easily recognize a defective product before purchase since pathogens are not detectable by sight or smell. After purchase, consumers cannot easily tie illness to the source of contamination—whether foodborne, and if so, which food and supplier. Similar to ‘credence goods’, consumers cannot easily substantiate the labeling or advertising food-safety claims of suppliers and industry-promoting regulatory agencies. As a result, consumers lack full market information that would allow them to confidently assess their willingness to pay for increased food safety, and given uncertain returns, suppliers lack clear profit incentives to incur the extra cost of providing more than minimum allowable levels of food safety.

An anonymous reviewer offered an alternative economic explanation for why beef outbreaks marginally increase in response to contractions in the cattle inventory not relying on market externalities. Producers may pay less attention to profits and management when contracting supply. In addition, consumers may pay less attention to the risk of foodborne illness when supply is short, and be misled by associated increased prices in presuming that higher-valued beef is safer.

Second, the annual cattle inventory (CI) drives live cattle futures prices (LCFprice), providing empirical evidence that traders have relied on CI as an key indicator of future supply conditions of live cattle as advised by commodity brokerage firms.

Perhaps surprising is the temporal regularity displayed by the above two causal interactions driven by CI when the multi-decadal cattle cycle isolated by SSA signal processing is below average. B-FDO predominantly marginally increases in response to increased CI when CI is contracted along the multi-decadal cycle. Of interest to traders, the marginal response of LCFprice to increased CI is predominantly positive when CI is contracted.

Cattle cycles occur systematically because producers, constrained by the reproductive biology of livestock, cannot instantly adjust inventories in response to price changes. Cattle cycles typically comprise three to four year ‘liquidation phases’ when cattle are sold off, and six to eight year ‘accumulation phases’ when herds are rebuilt. Indeed, SSA isolated cycles in cattle inventories of approximately six to nine years, which combined to explain 23% of total variation. In addition, SSA detected that these higher-frequency cattle cycles follow a stronger multi-decadal nonlinear trend cycle of about thirty years, accounting for almost 60% of total variation.

Third, B-FDO drives LCFprice, providing empirical evidence of agricultural market response to foodborne disease outbreaks that was expected but largely undetected in past studies. Confirming conjectures by Lusk and Schroeder (2002), the market reaction in our study was cumulative and endogenized into systematic long-term beef market dynamics. The market response is predominantly positive when the multi-decadal standardized B-FDO cycle isolated by SSA signal processing is below average (1980–1997), and otherwise more erratic. Past studies expected negative net price response on the basis that negative price impacts of downward shifts in demand for contaminated food would dominate positive price impacts of inward shifts in supply; however, the positive net price responses that we computed were also deemed possible [2].

## Concluding comments

We conclude with a broad caveat. Although we successfully reconstructed deterministic nonlinear dynamics from observational data in this case study, we cannot reasonably expect to do so in every application. Most obviously, the dynamics of a real-world system might not evolve along a low-dimensional nonlinear attractor. Moreover, available data may not adequately sample an existing real-world attractor. In general, we cannot reasonably expect to have observational data of sufficient quality to reconstruct the complex folding and fractal patterns of a real-world attractor [50]. We can reasonably hope to reconstruct the ‘sampling’ or ‘skeleton’ of a real-world attractor [51] if available data are long enough to represent the dominant time scales of the system, or not too noisy to detect deterministic behavior.

We recommend that food safety modelers take advantage of NLTS to initially test for systematic dynamic behavior in observed data before formulating policy models that fail to reflect this valuable information. NLTS data analytics provide a reliable empirical benchmark to guide specification and testing of new theoretical framings at the convergence of natural and economic sciences. This benchmark includes a geometric picture of real-world state space dynamics that models should be able to reproduce, an estimate of the minimum model dimensionality required, and identification of relevant model covariates and their interactions over time.

### Surrogate data

Surrogate data testing [19, 25] proceeds in three steps: First, null hypotheses are formulated to test for stochastic dynamic structures in a time series, and surrogate data vectors are computed compatible with these hypotheses. Surrogates destroy the serial structure of the time series while preserving statistical properties compatible with the null hypothesis. We computed *PPS* surrogates with an algorithm formulated by Small and Tse (2002), which test for noisy linear dynamics in cyclic time-series records. Second, a shadow attractor is reconstructed from each surrogate, and a discriminating statistic is computed measuring a hallmark of nonlinear deterministic behavior in each surrogate attractor. We selected nonlinear prediction skill [43] as a statistic that could be reliably computed from our data, and specified an upper-tailed test since deterministic-nonlinear structure is consistent with high nonlinear prediction skill, measured by the Nash-Sutcliffe Model Efficiency (NSE) [52]. Third, applying rank-order statistics [39], an ensemble of *S* = (*k*/*α*)−1 surrogates is generated, where *α* is the probability of false rejection and *k* controls the number of surrogates and consequently the sensitivity of the test. Setting *α* = 0.05 and *k* = 20, we accepted the null hypothesis of linear stochastic dynamics if the NSE taken from the shadow attractor reconstructed from the time series did not fall in the upper *k* values of NSE statistics taken from the ensemble of *S* = 399 surrogate attractors. If the null hypothesis is rejected, untested dynamic structures (i.e., nonlinear-deterministic dynamics) remain viable.

### Convergent cross mapping

CCM [9] finds that *X* is driven by *Y* if the shadow attractor reconstructed from *X* (*M*_*X*_) can skillfully cross-predict values of *Y*. The goodness-of-fit between cross-predicted and actual values of *Y* is measured with the Pearson correlation coefficient (*ρ*). Convergence in CCM requires that cross prediction become more skillful (i.e., *ρ* converges to an acceptable level) as the portion of the record for *X* used to reconstruct *M*_*X*_ (the *library*) increases in length; in other words, as the structural information in *M*_*X*_ increases. We further applied Extended-CCM [40] to screen skillful cross mappings for false positives in which synchronized behavior is mistaken for causal interaction [40]. Extended-CCM performs cross mappings over a spectrum of negative and positive delayed responses between driving variable *Y* and response variable *X*. If *Y* truly drives *X*, CCM performs best for a non-positive delayed response since true causality requires that causal events occur before (or contemporaneously with) provoked responses. Finally, we screened cross mappings for statistical significance by testing whether a shadow attractor *M*_*X*_ reconstructed from response variable *X* cross-predicts the potential driving variable *Y* with higher skill than it cross-predicts an ensemble of randomized PPS surrogate data vectors [41] constructed from *Y*. We ran CCM for *S* = (*k*/*α*)−1 = 399 surrogate data vectors (*k* = 20 and *α* = 0.05) and rejected the null hypothesis that *Y* is not a significant driver of *X* if the corresponding correlation coefficient was among the top *k* = 20 values computed with surrogate data vectors over a convergent subset of libraries. The floor of these upper surrogate correlation coefficients is portrayed graphically as a bootstrapped 95% confidence interval that a significant cross mapping curve exceeds.

### S-Mapping

S-Mapping [21] quantifies interactions identified with CCM. To compute the marginal response of *X* to *Y*, we first reconstructed a shadow attractor with phase space coordinates including *X* and *Y*, and built in the delayed response detected with Extended-CCM. S-Mapping computes the curvature of phase space at each point on a shadow attractor with a locally-weighted multivariate linear regression scheme. Estimated regression coefficients measure slopes in the direction of each coordinate variable at each point, and these slopes serve as partial derivatives of the response variable with respect to the driving variables in each time period.

### Code availability

The following R packages were used: RSSA (singular spectrum analysis); spacetime (space-time separation plots); tseriesChaos (mutual information function, false nearest neighbors test, time-delay embedding); multispatialCCM (convergent cross mapping); and igraph (causal network diagrams). These packages are downloaded from https://cran.r-project.org/package=*, where * is a package listed above. Wrap-around R code facilitating the use of these packages is available in Huffaker et al. (2017). R code to run the S-Mapping causality quantification algorithm is provided by Deyle et al. (2018). We used Origin 2020 [53] graphics software for 3-D plotting in Fig 3.

## Supporting information

S1 Appendix NLTS methods. (DOCX)
