# Supplementary material for: Reconstructing dynamics of foodborne disease outbreaks in the US cattle market from monitoring data
Source: PLoS One. 2021 Jan 27;16(1):e0245867. doi: 10.1371/journal.pone.0245867 (PMC7840002; doi:10.1371/journal.pone.0245867)
Supplement: S1 Appendix — (DOCX) [file pone.0245867.s001.docx]

**S1 Appendix. NLTS methods.**

**Singular Spectrum Analysis**

Singular Spectrum Analysis **(**SSA) proceeds in three steps: decomposition, grouping, and reconstruction [36]. In decomposition, a time series *F* of length *N* is embedded into an *L* x *K* trajectory matrix, *X*, whose *K* = *N* - *L* + 1 columns consist of single-period lagged vectors of *F*. Row dimension *L* is the preset ‘window length’ parameter determining the resolution of the decomposition, where 2 < *L* < *N*/2. The singular value decomposition (SVD) is taken of trajectory matrix, *X*, by computing the eigensystem of the *L* x *L* lagged covariance matrix

*S* = *XX* ^T^. The trajectory matrix is decomposed into the sum of new *L* x *K* matrices *X_i_* , where$X_{i}=\sqrt{\lambda_{i}}*V_{i}*EV_{i}^{T}, i = 1,...,K$, and *EV_i_* and *V_i_* are the eigenvector and left-eigenvector corresponding to eigenvalue λ*_i_*. In grouping, new matrices *X_i_* are summed into distinct groups forming the basis for trend, oscillatory, and unstructured residual components relying on diagnostic methods including scree plots of eigenvalues, eigenvector plots, and weighted correlation matrices [36]. In reconstruction, diagonal averaging converts each matrix group into a vector time series of a corresponding trend, oscillatory, or unstructured residual component.

**Singular Spectrum Transformation**

Singular Spectrum Transformation (SST) is implemented by running SSA in a sliding window *L*-periods wide through the signal [52, 53]. Each window is centered around a reference time that partitions the signal into non-overlapping past and future series of equal duration. SSA is run on the past and future series independently, and SST computes a change-point score (CP-score) indicating whether signal decomposition structurally changed within the window. SST produces a curve of CP-scores over the feasible range of reference times. In general, *L* must be sufficiently wide to provide for reliable signal decomposition of past and future series, and narrow enough to compute CP-scores at an informative time interval. The statistical significance of CP-scores is ascertained by bootstrapping an upper 90% confidence limit using randomized surrogate data vectors [52]. CP-scores resting above the upper confidence limit are statistically significant.

**Nonlinear phase space reconstruction**

Time-delay embedding reconstructs shadow phase space from a single record *x*(*t*) with delayed copies *x*(*t+d*), *x*(*t+2d*),… *x*(*t+md*) serving as phase space surrogates for omitted variables, where *d* is the embedding delay, and the number of delayed embedding coordinates is the embedding dimension *m*. Embedding delay *d* is conventionally estimated as the first minimum of the average mutual information function [19]. Takens (1980) proved that a shadow attractor provides a one-to-one mapping to the original attractor if *m* is sufficiently large to contain the original attractor [24]. Embedding dimension *m* is calculated with the false-nearest-neighbors test, which identifies the dimension in which neighboring points on the shadow attractor cease to diverge substantially [19]. The false-nearest-neighbors test requires that possible serial correlation in the data be corrected to avoid mistaking temporal proximity between points on a shadow attractor for geometric structure. Points within the temporal proximity of the Theiler window [54] are deemed to be serially correlated, and consequently disqualified as nearest neighbors to a given reference point.

The Theiler window is estimated with space-time separation plots [55], which scatterplot the spatial distance (vertical axis) and elapsed time (horizontal axis) between each pair of points in *shadow* phase space. This information is conventionally reformatted as equal-probability contour lines by plotting the percentage of pairs that are less than or equal to a given distance, and drawing curves through identical percentages across values of time. In the simplest plots, contours increase at a decreasing rate toward a saturation distance $\varepsilon$(vertical axis), and the Theiler window is selected as the elapsed time $\Delta t$(horizontal axis) at which saturation commences. In more complex plots, contours cycle, and the Theiler window is selected as the $\Delta t$ after which contours cease to increase consistently in the initial cycle [19]. Contours continuing to rise indicate that temporal distance between points never ceases to affect their spatial distance, and phase space reconstruction fails. Fig A1 in the Appendix shows the space-time separation plots for B-FDO, CI, and LCFprice, from which we estimated Theiler windows of 10, 15, and 20 years, respectively.





**Fig A1. Space-time separation plots.** Theiler windows are estimated with space-time separation plots made up of contour lines plotting the percentage of paired points on a shadow attractor that are less than or equal to a given distance (vertical axis) across time (horizontal axis). When contours cycle, the Theiler window is selected as the elapsed time after which contours cease to increase consistently in the initial cycle. Figure A1 shows the space-time separation plots for B-FDO, CI, and LCFprice, from which we estimated Theiler windows of 10, 15, and 20 years, respectively.

**References**

52. Itoh N, Marwan N. An extended singular spectrum analysis transformation (SST) for the investigation of Kenyan precipitation data. Nonlinear Processes in Geophysics. 2013;20:467-81.

53. Moskvina V, Zhigljavsky A. An algorithm based on singular spectrum analysis for change-point detection. Communications in Statistics. 2003;32:319-53.

54. Theiler J. Spurious dimension from correlation algorithms applied to limited time series data. Phys Rev A. 1986;34:2427-32.

55. Provenzale A, Smith L, Vio R, Murante G. Distinguishing between low-dimensional dynamics and randomness in measured time series. Physica D. 1992;58:31.
